# Motor imagery of gait: a new way to detect mild cognitive impairment?

**DOI:** 10.1186/1743-0003-11-66

**Published:** 2014-04-18

**Authors:** Olivier Beauchet, Cyrille P Launay, Ervin Sejdić, Gilles Allali, Cédric Annweiler

**Affiliations:** 1 Department of Neuroscience, Division of Geriatric Medicine, Angers University Hospital, 49933 Angers cedex 9, France; 2 Biomathics, Angers, France; 3 Department of Electrical and Computer Engineering, Swanson School of Engineering, University of Pittsburgh, Pittsburgh, USA; 4 Department of Neurology, Geneva University Hospital and University of Geneva, Geneva, Switzerland; 5 Robarts Research Institute, Department of Medical Biophysics, Schulich School of Medicine and Dentistry, the University of Western Ontario, London, ON, Canada

**Keywords:** Gait, Motor imagery, Mild cognitive impairment, Alzheimer disease

## Abstract

**Objectives:**

1) To measure and compare the time required to perform (pTUG) and the time required to imagine (iTUG) the Timed Up & Go (TUG), and the time difference between these two tasks (i.e., TUG delta time) in older adults with cognitive decline (i.e., mild cognitive impairment (MCI) and mild-to-moderate Alzheimer disease and related disorders (ADRD)) and in cognitively healthy individuals (CHI); and 2) to examine any association between the TUG delta time and a cognitive status.

**Methods:**

Sixty-six participants (24 CHI, 23 individuals with MCI, and 19 individuals with ADRD) were recruited in this cross-sectional study. The mean and standard deviation of the pTUG and iTUG completion times and the TUG delta time, as well as age, gender, and Mini-Mental State Examination (MMSE) scores were used as outcomes. Participants were separated into three groups based on the tertilization of TUG delta time: lowest (<13.6%; n = 22; best performance), intermediate (13.6-52.2%; n = 22), and highest tertile (>52.2%; n = 22, worst performance).

**Results:**

Fewer CHI were in the group exhibiting the highest tertile of TUG delta time compared to individuals with lowest and intermediate TUG delta times (p = 0.013). Being in the highest tertile of the TUG delta time was associated with cognitive decline in the unadjusted model (p = 0.012 for MCI, and p = 0.021 for mild-to-moderate ADRD). In the multivariate models, this association remained significant only for individuals with MCI (p = 0.019 while adjusting for age and gender; p = 0.047 while adjusting for age, gender, and MMSE score; p = 0.012 for the stepwise backward model).

**Conclusions:**

Our results provide the first evidence that motor imagery of gait may be used as a biomarker of MCI in older adults.

## Background

There is growing evidence that cognitive decline results in the deterioration of gait patterns [1-3]. Though it is commonly associated with the later stages of dementia, a decline in gait performance may also be detected much earlier. Some go so far as to suggest that this decline can be detected before the prodromal stage of mild cognitive impairment (MCI) [1-8]. In either case, there is an obvious association between gait disorders and the impairment of the highest levels of gait control [4-8].

The diagnosis of MCI is based on a comprehensive neuropsychological assessment which explores various cognitive domains, such as episodic memory and executive function, combined with blood tests and brain imaging [1-5]. However, this diagnostic process is time-consuming, expensive and does not conclusively demonstrate that an individual has MCI. Since interventions appear to be more effective in early stages of the disease, improving early diagnosis of dementia at the prodromal stage of MCI is an important research goal. Recently, the use of biomarkers has been proposed to facilitate the early diagnosis of dementia [9]. Biomarkers are defined as indicators of a disease process, and their complementary use to classical neuropsychological tools is essential to this aim [9]. For example, specific proteins in the cerebrospinal fluid (CSF) (e.g. protein tau) indicate the presence of Alzheimer disease (AD) and so serve as biomarkers when screening for that condition [9]. However, the main limitation of CSF biomarkers is the compulsory invasive examination, often referred to as CSF tapping. If a clear link can be established between spatio-temporal gait parameters and the motor disorders of early-stage dementia, an alternative, non-invasive biomarker could be implemented. Such a biomarker could be more easily assessed in clinical practice and could serve to improve the prediction accuracy of these screening procedures, particularly those related to AD [10].

Motor imagery is the mental simulation of a given action without its actual execution. It is a method used clinically to explore the highest level of gait control [11,12]. Recently, a mental chronometry approach was used to show that cognitive performance, executive functioning in particular, contributes to the temporal correspondence between executing and imaging gait in patients with dementia, schizophrenia, and multiple sclerosis [13-15]. This approach showed that older adults with cognitive decline executed the imagined Timed Up & Go test (iTUG) more quickly than the same task actually performed (pTUG), although this was not the case in healthy younger adults [11]. The pTUG test offers a basic assessment of a patient’s functional mobility by measuring the time while standing up, walking, turning, and sitting down. This test has been used to evaluate gait and balance performance in previous studies/the clinical environment/wherever [16].

Similar to gait motor imagery, gait variability also reflects the functionality of the highest level of gait control. Higher gait variability was reported for individuals with MCI but not for individuals with AD [2]. This suggests that higher gait variability in individuals with MCI may reflect an early dysfunction of the cortical sensorimotor control of gait involving the hippocampus [15,2]. Therefore, we hypothesized that a poor time correspondence between pTUG and iTUG completion times will be observed in individuals with MCI rather than in those with ADRD or in cognitively healthy individuals (CHI). This conclusion was reached because individuals with MCI are suspected to have more difficulties imagining gait than actually walking. The objectives of this cross-sectional study were 1) to measure and to compare pTUG and iTUG completion times as well as the time difference between these two motor tasks (i.e., TUG delta time) in older adults with and without cognitive decline; and 2) to examine whether there was an association between the TUG delta time and the cognitive status (e.g., CHI, MCI, ADRD).

## Methods

### Participants

From December 2009 until November 2010, 66 participants (24 CHI [mean age 68.8 ± 4.6 years; mean Mini-Mental State Examination score (MMSE) score 29.0 ± 1.2; 79.2% female], 23 individuals with MCI [mean age 70.8 ± 4.6 years; MMSE score 27.8 ± 1.3; 65.2% female] and 19 individuals with mild-to-moderate ADRD [mean age 80.1 ± 4.3 years; MMSE score 20.4 ± 4.7; 89.5% female]) were recruited in the "GAIT" (Gait and Alzheimer Interaction Tracking) study, which is a cross-sectional study designed to compare gait characteristics of CHI and participants with MCI and ADRD. The data collection procedure has been described elsewhere in detail [2,17]. In brief, all participants were referred for evaluation of memory complaints at the memory clinic of Angers University Hospital, France. The eligibility criteria were: age 65 years and over, ambulatory, an adequate understanding of French, and no history of acute medical illness in the past month. For the present analysis, exclusion criteria were: severe ADRD (i.e., MMSE ≤ 9 [18]), extrapyramidal rigidity of the upper limbs, neurological and psychiatric diseases other than cognitive impairment, severe medical conditions affecting walking, inability to walk 15 minutes unassisted, or the presence of depressive symptoms defined by a 4-item Geriatric Depression Scale score above 1 [19].

Participants in the study were included after having given their written informed consent for research. The study was conducted in accordance with the ethical standards set forth in the Helsinki Declaration (1983) and the entire study protocol was approved by the local Ethical Committee of Angers, France.

### Neuropsychological and physical assessment

A neuropsychological assessment was performed on each participant during a face-to-face examination by a neuropsychologist. The following standardized tests were used to probe several aspects of cognitive function: MMSE [18], Frontal Assessment Battery (FAB) [20], Alzheimer's Disease Assessment Scale–Cognitive subscale (ADAS-cog) [21], Trail Making Test (TMT) parts A and B [22], French version of the Free and Cued Selective Reminding Test [23,24], and Instrumental Activities of Daily Living scale (IADL) [25]. The diagnoses of MCI and ADRD were made during multidisciplinary meetings involving geriatricians, neurologists, and neuropsychologists of Angers University Memory Clinic, and they were based on the aforementioned neuropsychological tests, physical examination findings, blood tests, and brain magnetic resonance imaging (MRI). MCI was diagnosed according to the criteria detailed by Winblad et al. [26]. Participants with any form of MCI, be it amnesic or non-amnesic and affecting single or multiple domains, were included in this study. The diagnosis of ADRD followed the DSM-IV and NINCDS/ADRDA criteria [27]. Mild-to-moderate stages of ADRD were defined by a MMSE score greater than or equal to 10. Participants who had neither MCI nor ADRD and who had normal neuropsychological and functional performances were considered as cognitively healthy.

### Gait assessment

This study used the TUG test described by Podsiadlo and Richardson [16]. Participants were asked to perform the TUG (pTUG) at their self-selected pace in a well-lit environment. Participants were permitted to use their walking aid if needed. All participants completed one trial of the pTUG and then one trial of the iTUG while sitting on a chair. Times for each condition were recorded with a stopwatch to the nearest 0.01 second. Before testing, a trained evaluator gave standardized verbal instructions regarding the test procedure. For the pTUG, participants were initially seated and were allowed to use the armrests to stand up if necessary. The pTUG participants were instructed to walk three meters, to turn around, to walk back to the chair, and lastly to sit down. For the iTUG, participants remained seated in the chair and were instructed to imagine doing the TUG and to say “stop” out loud when they were finished. Participants could choose to do the iTUG with their eyes opened or closed. The stopwatch was started on the command “ready-set-go” and stopped when the participant pronounced the word “stop”.

### Outcome variables

The main outcome variables were: 1) the mean ± SD of the time to completion for both the pTUG and iTUG; 2) the mean ± SD of the TUG delta time, which was calculated according to the following formula: [(pTUG– iTUG) / (pTUG + iTUG / 2)] x 100; 3) the mean ± SD of the participants’ age; 4) sex of the participants; 5) the cognitive status of the participants as either CHI, MCI, or mild-to-moderate ADRD; and 6) the global cognitive performance estimated by the mean ± SD of the MMSE score.

### Statistics

The participants’ characteristics were summarized using either mean ± SD or frequencies and percentages, as appropriate. Participants were separated into 3 groups based on the tertilization of the TUG delta time: lowest (<13.6%; n = 22; best performance), intermediate (13.6-52.2%; n = 22), and highest tertile (>52.2%; n = 22, worst performance). Between-group comparisons were performed using Kruskal-Wallis, Mann–Whitney, or Chi-square tests, as appropriate. Univariate and multiple logistic regression analyses were performed to specify the association between the worst tertiles of TUG (i.e., the highest tertile of TUG delta time, the highest tertile of pTUG [i.e., >10.9 seconds] and the lowest tertile of iTUG [i.e., <6.0 seconds]) used as dependent variables and the cognitive status (MCI and mild-to-moderate ADRD, compared to CHI as reference level) used as independent variables adjusted for baseline characteristics (i.e., age, gender and MMSE score). P-values that were less than 0.05 were considered statistically significant. All statistics were performed using SPSS (version 19.0; SPSS, Inc., Chicago, IL).

## Results

Comparisons among groups showed that individuals with the worst performance, defined as being in the highest tertile for TUG delta time, were older than those in the intermediate (p = 0.019) and lowest tertiles (p = 0.026) (Table 1). They had also a lower MMSE score compared to those in the intermediate tertile (p < 0.001) and those in lowest tertile (p = 0.016). There were fewer CHI in the highest tertile group than in the lowest (p = 0.014) or intermediate (p = 0.004) groups. In addition, individuals in the highest tertile group of TUG delta time had worse TUG performance compared to the lowest and the intermediate groups (all p-values < 0.006) across all of the examined criteria (i.e., pTUG, iTUG, and TUG delta time). Similar results were shown with the comparison between the lowest and the intermediate tertile groups (all p-values <0.001) except for the pTUG completion time, for which there was no difference (p = 0.354). As shown in Table 2, the logistic regression models indicated that the highest tertile of TUG delta time was associated with cognitive impairments in the unadjusted model (p = 0.012 for MCI, and p = 0.021 for ADRD). Adjusted models showed that this association remained significant only with the MCI status (p = 0.019 while adjusting for age and gender; p = 0.047 for the fully adjusted model; p = 0.012 for the stepwise backward model). Furthermore, the MMSE score was also associated with the highest tertile of TUG delta time in the stepwise backward logistic regression (p = 0.009). While considering the highest tertile of pTUG performance as a dependent variable, an association was found with mild-to-moderate ADRD (p = 0.021) in the unadjusted regression model, but it disappeared in the multivariate models. Stepwise backward modelling showed an association only with MMSE score (p = 0.002). Lastly, a significant association was shown between the lowest tertile of iTUG performance and MCI status in the stepwise backward model (p = 0.013).

**Table 1:** Characteristics of participants separated into 3 groups according to the tertiles of TUG delta time* (n = 66)

	**Tertiles of TUG delta time**			**P-Value**^**†**^			
	**Lowest (n = 22)**	**Intermediate (n = 22)**	**Highest (n = 22)**	**Overall**	**Lowest versus intermediate**	**Lowest versus highest**	**Intermediate versus highest**
Age (years), mean ± SD	71.4 ± 5.9	71.1 ± 6.4	76.3 ± 7.0	**0.028**	0.621	**0.026**	**0.019**
Female, n (%)	14 (63.6)	15 (68.2)	15 (68.2)	0.934	-	**-**	**-**
Cognitive status, n (%)							
CHI	10 (45.5)	12 (54.5)	2 (9.1)	**0.013**	0.650	**0.014**	**0.004**
MCI	6 (27.3)	7 (31.8)	10 (45.5)	0.173	-	**-**	**-**
Mild-to-moderate ADRD	5 (22.7)	3 (13.6)	6 (27.3)	0.335	-	**-**	**-**
MMSE score (/30), mean ± SD	26.5 ± 4.6	28.2 ± 2.3	23.6 ± 5.2	**0.001**	0.168	**0.016**	**<0.001**
Timed Up & Go test, mean ± SD							
pTUG (seconds)	9.8 ± 2.6	9.1 ± 1.6	15.0 ± 9.3	**<0.001**	0.354	**0.005**	**<0.001**
iTUG (seconds)	10.1 ± 2.9	6.6 ± 1.3	5.5 ± 1.9	**<0.001**	**<0.001**	**<0.001**	**0.018**
TUG Delta time* (%)	-2.3 ± 15.8	31.2 ± 11.1	84.9 ± 25.5	**<0.001**	**<0.001**	**<0.001**	**<0.001**

CHI: Cognitively healthy individual.

MCI: Mild cognitive impairment.

ADRD: Alzheimer disease and related disorders.

MMSE: Mini-Mental status examination.

TUG: Timed Up & Go test.

pTUG: performed TUG.

iTUG: imagined TUG.

n: number of participants.

*: Calculated from the formula: delta time = [(pTUG–iTUG)/(pTUG + iTUG)/2] ×100.

^†^: Between-group comparisons were performed using Kruskal-Wallis, Man-Whitney, or Chi-square tests, as appropriate.

P significant (<0.05) indicated in bold.

**Table 2:** Univariate and multiple logistic regression models showing the association between a) the highest tertile of TUG delta time*, b) the highest tertile of pTUG and c) the highest tertile of iTUG (dependent variables) and cognitive status (independent variable) adjusted for participants' characteristics (n = 66)

	**Model 1**			**Model 2**			**Model 3**			**Model 4**		
	**OR**	**95% CI**	**P-value**	**OR**	**95% CI**	**P-value**	**OR**	**95% CI**	**P-value**	**OR**	**95% CI**	**P-value**
**a) TUG delta time**												
CHI	1.00 (Ref^†^)			1.00 (Ref^†^)			1.00 (Ref^†^)					
MCI	8.5	[1.6;44.8]	**0.012**	8.6	[1.4;51.8]	**0.019**	6.8	[1.1;45.3]	**0.047**	5.9	[1.5;23.6]	**0.012**
Mild-to-moderate ADRD	8.3	[1.4;49.6]	**0.021**	2.8	[0.3;29.8]	0.394	0.4	[0.0;13.4]	0.594	**-**	**-**	**-**
Age				1.1	[1.0;1.3]	0.190	1.1	[1.0;1.3]	0.117	**-**	**-**	**-**
Female gender				1.4	[0.3;5.9]	0.635	1.3	[0.3;6.1]	0.769	**-**	**-**	**-**
MMSE							0.8	[0.6;1.1]	0.136	0.8	[0.6;0.9]	**0.009**
**b) pTUG**												
CHI	1.00 (Ref^†^)			1.00 (Ref^†^)			1.00 (Ref^†^)					
MCI	0.5	[0.1;2.1]	0.305	0.7	[0.7;2.1]	0.260	0.2	[0.0;1.4]	0.104	-	-	-
Mild-to-moderate ADRD	5.4	[1.3;22.6]	**0.021**	1.3	[0.2;10.4]	0.807	0.1	[0.0;3.0]	0.188	**-**	**-**	**-**
Age				0.8	[1.0;1.3]	0.0.75	1.2	[1.0;1.4]	0.055	**-**	**-**	**-**
Female gender				1.5	[0.3;6.8]	0.611	1.2	[0.2;6.1]	0.804	**-**	**-**	**-**
MMSE							0.7	[0.5;1.0]	**0.049**	0.7	[0.6;0.9]	**0.002**
**c) iTUG**												
CHI	1.00 (Ref^†^)			1.00 (Ref^†^)			1.00 (Ref^†^)					
MCI	3.5	[1.0;12.5]	0.056	4.1	[0.9;18.3]	0.060	4.1	[0.8;20.3]	0.086	4.4	[1.4;14.3]	**0.013**
Mild-to-moderate ADRD	0.6	[0.1;3.8]	0.617	0.9	[0.1;9.1]	0.900	0.3	[0.0;12.0]	0.541	**-**	**-**	**-**
Age				0.7	[0.9;1.1]	0.973	1.0	[0.9;1.1]	0.984	**-**	**-**	**-**
Female gender				1.3	[0.3;5.1]	0.718	1.4	[0.3;6.0]	0.646	**-**	**-**	**-**
MMSE							0.9	[0.6;1.2]	0.497	**-**	**-**	**-**

CHI: Cognitively healthy individual.

MCI: Mild cognitive impairment.

ADRD: Alzheimer disease and related disorders.

MMSE: Mini-Mental status examination.

TUG: Timed Up & Go test.

pTUG: performed TUG

iTUG: imagined TUG

OR: odds ratio.

CI: confidence interval.

n: number of participants.

Model 1: Unadjusted model.

Model 2: Adjusted on age and gender.

Model 3: Adjusted on all participants' characteristics.

Model 4: Stepwise backward model.

*: Calculated from the formula: delta time = [(pTUG–iTUG)/(pTUG + iTUG)/2] x100 with pTUG = performed TUG, and iTUG = imagined TUG.

^†^: CHI used as reference level.

P-value significant (<0.05) indicated in bold.

## Discussion

The main finding of this study is that individuals with MCI executed the iTUG trial faster than the pTUG trial in comparison to individuals with CHI.

Our findings are in concordance with a previous study showing that cognitive decline was related to the time difference between the pTUG and iTUG completion times [13]. In comparison to this previous study, we examined the effects of early cognitive decline (i.e., MCI and mild-to-moderate ADRD) using a mental chronometry approach. The association found between the worst (i.e., highest) TUG delta time and the MCI status confirmed the fact that abnormal gait control estimated from pTUG and iTUG trials should be considered as an early biomarker of dementia, as suggested in two recently published studies [2,3]. The first report outlined an association between MCI status and higher gait variability [2]. Gait variability is defined as fluctuations in stride-to-stride intervals and reflects the cortical control of gait [4-8]. Increased gait variability reported in demented individuals is usually interpreted as abnormal gait control provoked by cognitive impairment [4,6,8]. Using a similar study design and the same groups of participants, the increased gait variability observed of individuals in a past study with MCI was explained by cortical cognitive dysfunction with subsequent cortical misprocessing of sensorimotor information [2]. These factors combined to produce higher gait variability [2]. Verghese et al. [3], showed that the combination of memory complaints with slower gait, defined as the Motor Cognitive Risk (MCR) syndrome, was predictive of a higher risk of dementia. This study provided preliminary support for a motor-based MCR syndrome that could identify older individuals at high risk for transitioning to dementia. The result of the present study agreed with these two prior reports [2,3]. Indeed, the high TUG delta time exhibited by individuals with MCI could be considered as an early biomarker of cognitive decline, similar to high gait variability for the MCR syndrome. In this study, however, we used a new and complementary approach based on mental chronometry that is designed to be implemented in clinical practice. Using functional magnetic resonance imaging, previous studies showed that motor imagery of gait activated several brain areas including the supplementary motor area, the bilateral precentral gyrus, the left dorsal premotor cortex, and the cingulated motor area [28-30]. The specific involvement of the hippocampus was also recently found in an fMRI study comparing mental imagery of gait between young and older adults [31]. Different brain areas such as the prefrontal cortex, Brodmann area 6 and the posterior supplementary motor cortex in particular, seem to be predominantly related to the MCI status [28,30,31]. Following the previous observations, it can be postulated that individuals with MCI may also have deficits in those regions which may disturb their motor imagery ability. The latter point could explain the positive association between MCI status and delta time reported in our study.

Our findings also highlight the fact that MMSE score is inversely related to change in TUG delta time, which is in accordance with a previous study [2]. It should also be noted that the high delta time was specifically related to the MCI status, even though ADRD participants with mild-to-moderate dementia had a lower MMSE score compared to the other groups. One explanation is based on the fact that individuals with moderate dementia experience difficulties walking, while those with MCI may have more difficulties imagining gait than actually performing the task. This would suggest a lack of a dose–response between cognitive decline and motor imagery, which would explain why only the stage of MCI may be detected with TUG delta time. An alternative explanation could be that cognitive functions, and consequently motor imagery ability, are too impaired in people with dementia to perform the iTUG trial correctly. Thus, the performance of iTUG trial is not readily interpretable and thus not suitable for detecting the diagnosis of ADRD. In contrast with the previous study, we did not find an effect of age on the TUG delta time. There are two main explanations: first, we focused only on older adults unlike the previous study that examined younger and older adults. Second, it is now well-recognized that participants with cognitive decline present greater gait impairments than those that occur naturally through aging [1,3,15].

Our study has some limitations. First, its cross-sectional design may limit the exploration of the association between TUG performance and cognitive disorders. Second, residual potential confounders may still be present even though we were able to control many characteristics likely to modify this association.

## Conclusion

These results provide the first evidence that the iTUG test, which was designed to assess motor imagery of gait, can be used as a biomarker of MCI in older adults.

## Competing interest

The authors report no conflicts of interest.

## Authors’ contributions

OB has full access to the data in the study and takes responsibility for the integrity of the data and the accuracy of the data analyses; study concept and design: OB and CA; acquisition of data: CL and GA; analysis and interpretation of data: OB, CPL, GA and CA; drafting of the manuscript: OB and CPL; critical revision of the manuscript for important intellectual content: ES, GA and CA; obtained funding: OB; statistical expertise: OB; administrative, technical, or material support: OB and CA; study supervision: OB and CA. All the authors (OB, CPL, ES, GA, CA) have participated in the research reported, have seen and approved the final version of the manuscript, and have agreed to be an author of the paper.
